# Early Onset of Autoimmune Diabetes in Children with Down Syndrome—Two Separate Aetiologies or an Immune System Pre-Programmed for Autoimmunity?

**DOI:** 10.1007/s11892-020-01318-8

**Published:** 2020-08-25

**Authors:** Georgina L. Mortimer, Kathleen M. Gillespie

**Affiliations:** Diabetes and Metabolism, Bristol Medical School, Level 2, Learning and Research, Southmead Hospital, University of Bristol, Bristol, BS10 5NB UK

**Keywords:** Down syndrome, Type 1 diabetes (T1D), Autoimmunity, Islet autoantibodies, HLA

## Abstract

**Purpose of Review:**

An increased frequency of autoimmunity in children with Down syndrome (DS) is well described but few studies have investigated the underlying mechanisms. Recent immune system investigation of individuals with DS may shed light on the increased risk of autoimmune conditions including type 1 diabetes.

**Recent Findings:**

Diagnosis of type 1 diabetes is accelerated in children with DS with 17% diagnosed at, or under, the age of 2 years compared with only 4% in the same age group in the general population. Counterintuitively, children with DS and diabetes have less human leukocyte antigen (HLA)-mediated susceptibility than age-matched children with autoimmune diabetes from the general population. Early onset of diabetes in DS is further highlighted by the recent description of neonatal cases of diabetes which is autoimmune but not HLA associated. There are two potential explanations for this accelerated onset: (1) an additional chromosome 21 increases the genetic and immunological risk of autoimmune diabetes or (2) there are two separate aetiologies in children with DS and diabetes.

**Summary:**

Autoimmunity in DS is an under-investigated area. In this review, we will draw on recent mechanistic studies in individuals with DS which shed some light on the increased risk of autoimmunity in children with DS and consider the current support for and against two aetiologies underlying diabetes in children with DS.

## Introduction

Therapeutic strategies targeting the underlying mechanisms of autoimmunity may prove of great clinical value: a recent study demonstrated that autoimmune disease was the underlying or contributory cause in 3.4% of deaths in females after the first year of life [1]. It is clear that common mechanisms underlie these diseases: genome-wide association studies (GWAS) emphasise the importance of the human leukocyte antigen (HLA) and suggest roles for several T cell suppressor genes, yet mechanistic data are limited. Immunological insights into susceptibility to autoimmunity are increasing from an unexpected avenue, autoimmune pathways in children with Down syndrome (DS).

DS is a relatively common condition affecting over 300,000 people in the USA and 30,000 in the UK and ranging from 1:700 to 1:1000 live births [2, 3]. Those born with DS have increased risk of many complex health conditions including congenital heart defects, leukaemia, dementia and autoimmune conditions [4].

### Autoimmunity in DS

Hypothyroidism is present in one-third of children with DS (although some may result from thyroid hypoplasia) [5] and celiac disease-associated antibodies in 10% [6, 7]. A considerably increased risk of developing clinically diagnosed type 1 diabetes (T1D) has been consistently reported in children with DS [8–11]. In a questionnaire-based study of 20,362 patients with DS in the UK and USA, the prevalence of diabetes diagnosed before age 20 in the DS population was 6 times higher than expected [8]. A more recent population-based study of the prevalence of T1D in DS in Denmark demonstrated a 4-fold increased risk of T1D in children with DS [12]. In these studies of diabetes in DS, no attempts were made to systematically characterise diabetes in DS.

### Autoimmune Diabetes in DS

Autoantibodies to islet antigens, insulin (IAA) [13], glutamate decarboxylase (GADA) [14], islet antigen-2 (IA-2A) [15, 16] and zinc transporter 8 (ZnT8A) [17] and more recently tetraspanin-7 [18] are the most reliable markers of humoral activity in autoimmune diabetes and combinations of two or more islet autoantibodies remain the best predictors of the condition [19]. As islet autoimmunity evolves, there is typically spreading of reactivity to different target autoantigens and epitopes. IAA are often the first autoantibodies to be detected in infancy, followed by GADA [20, 21], while IA-2A and ZnT8A tend to appear later.

To determine whether islet autoantibodies are more common in children with DS compared with the general population, we analysed the frequency of T1D-associated islet autoantibodies in 106 children with DS and found evidence of 2 or more antibodies in 6/106 (5.7%) compared to 0.28% in an age-matched control population (*p* < 0.001). Intriguingly, the titres of antibodies to GADA were particularly high in some of these children [22•].

These data provide immunological evidence that autoimmune diabetes is increased in children with DS. It has been suggested that diabetes in DS generally presents early in life; one study from the 1960s showed a peak onset at 8 years of age, compared with 14 years in contemporary cases of childhood diabetes [23]. In a more recent study of DS and T1D by the authors, 22% had developed diabetes by the age of 2 years, as compared to 7% of those from the general diabetes population [24••]. This indicates that onset is accelerated in DS children compared with children with T1D in the general population. Concerns that this pattern was caused by selection bias were alleviated by a study from the Diabetes-Patienten-Verlaufsdaten, a longitudinal follow-up database, which collects data from 298 German and Austrian diabetes centres [25]. Their data from over 42,000 children with diabetes also showed a biphasic pattern in age at onset in 159 children with DS and diabetes. Of interest is the observation in this study that children with DS and diabetes used less insulin but showed better glycaemic control. Many studies of those diagnosed with DS and diabetes aimed to calculate the frequency of both conditions (and other forms of autoimmunity) co-occurring. A list of published populations is outlined in Table 1.

**Table 1:** Studies of diabetes and autoimmunity in D.S

**Reference**	**Year**	**Country**	**DS and T1D (n= )**	**Age at Diagnosis (years) mean/median as published**	**Findings**
Milunsky and Neurath [8]	1968	U.K. and U.S.	88		High prevalence of T1D in DS
Rabinowe et al [30]	1989	U.S.	3		Islet and thyroid autoantibodies in DS, T cell abnormalities
Griffin et al [31]	1997	Taiwan	6		Thyroid autoantibodies and function in DS
Anwar et al [11]	1998	U.K.	13	22	Simpler insulin regimens but improved glycaemic control
Shield et al [32]	1999	U.K.	16	6.7	Earlier age at diagnosis, no increased disomic homozygosity in the region of the autoimmune polyglandular syndrome type 1 locus
Aktay et al [33]	2001	U.S.	2		Celiac autoantibodies in T1D population, some had DS
Makovi et al [34]	2002	Hungary	2		Childhood diabetes in population, some of which had DS
Bergholdt et al [12]	2006	Denmark	8	6	Increases prevalence of DS in T1D population, HLA Genotyping showed some at risk and one protective, positive islet autoantibodies where measured, co-occurring multiple autoimmunity
Gillespie et al [22]	2006	U.K.	40	9	Increased Islet autoantibodies in DS children, Islet autoantibody profile in DSD cohort, HLA risk in DSD increased but lowered high-risk compared to T1D
Rohrer et al [25]	2010	Germany and Austria (DPV)	159	8.2 ± 5.3	DSD less frequent insulin and improved glycaemic control, younger age at onset, increased Celiac and Thyroid autoantibodies
Kota et al [35]	2012	India	2	10.5 ± 7.2	Varied clinical profiles of co-existing conditions in T1D population, some of which had DS
Schmidt et al [36]	2012	Germany and Austria (DPV)	141	8.32 ± 4.2	Several genetic syndromes associated with T1D, islet autoantibodies were positive in DS, Turner syndrome and Friedreich ataxia
Aitken et al [24]	2013	U.K.	136		Age at onset is lower in DSD and biphasic, all diagnosed under 2 were GADA positive, co-occurring multiple autoimmunity
Taggart et al [37]	2013	Northern Ireland	42		T1D in intellectual disability more prevalent in DS and autism spectrum disorder
Aversa et al [38]	2016	Italy	7		Co-occurring multiple autoimmunity with autoimmune thyroid diseases, association with DS
Abdulrazzaq et al [39]	2018	U.A.E	4		Co-occurring multiple autoimmunity in DS, positive islet, thyroid and celiac autoantibody profiles, celiac is less prevalent than T1D and thyroid
Johnson et al [26]	2019	U.K. and U.S.	13	Neonatal	DS is a cause of autoimmune neonatal diabetes, but it is not associated with T1D polygenic risk

### Neonatal Diabetes in Children with DS

Johnson and colleagues [26••] observed that among a unique cohort of 1522 children diagnosed with permanent neonatal diabetes diagnosed before the age of 6 months, 13 (0.9%) had Down syndrome, some 6.7-fold higher than the expected population frequency of 12.6 per 10,000. None of the children tested positive for recognised mutations for neonatal diabetes. Samples were available for islet autoantibody testing on 9 participants with time from diagnosis ranging from 4 months to 10 years. Of these, four were positive for GADA. Using a polygenic T1D risk score dominated by HLA-mediated risk [27], DS cases with neonatal diabetes did not have increased risk. In addition, four of the 13 (31%) were positive for the protective HLA DR15-DQB1*0602 haplotype which is observed in only 1% of type 1 diabetes cases in the general population.

Heterogeneity of diabetes in DS is supported by genetic studies. The frequency of diabetes-associated high-risk HLA haplotypes in children with DS and T1D (diagnosed before the age of 21) was examined [24••]. There was an excess of diabetes-associated HLA class II genotypes in children with DS and T1D compared to age and sex-matched healthy controls (*p* < 0.001) indicating that, as might be expected, autoimmune diabetes in DS shares the same HLA susceptibility as T1D in the general population. DS children with T1D were however less likely to carry the highest-risk genotype DR4-DQ8/DR3-DQ2 than children with T1D from the general population (*p* = 0.01) and more likely to carry low-risk (DR2-DQ6/X or X/X) genotypes (*p* < 0.0001).

In summary, therefore, clinical diagnosis of diabetes in children with DS appears accelerated compared with the general population. In type 1 diabetes, children who develop the condition in early life (for instance under the age of 5 years) have increased HLA-mediated susceptibility [28]. Why do children with DS and diabetes have less HLA risk? One possible explanation is that two different aetiologies are underlying the increased prevalence of diabetes in DS; one HLA mediated and one which is not. Larger cohorts of individuals with DS and diabetes with samples available at diagnosis for islet autoantibody screening are required to address this question.

### Pancreatic Pathology in DS and Diabetes

Studies of the pancreas in individuals with DS and diabetes are extremely rare. In the seminal study of pancreatic pathology in type 1 diabetes, however, Foulis et al. described three cases of DS and diabetes [29]. One was a 14-year-old boy who had diabetes for several years and another, a 12-year-old with recent-onset diabetes: both showed evidence of lymphocytic infiltration with the absence of insulin staining in the 14-year-old boy, typical of T1D. The third child, however, diagnosed with diabetes at 18 months whose pancreas was analysed within 2 weeks of diagnosis of diabetes displayed normal insulin staining with no morphological abnormality. This might add evidence to the suggestion that there are two aetiologies of diabetes in children with DS.

Butler and colleagues investigated whether the increased frequency of diabetes in DS was due to congenital defects in beta cells. Autopsy pancreas from individuals with DS and age-matched control subjects were examined and no difference was observed in the fractional islet beta-cell area between the two groups [40].

### Immune Development in DS

Immune dysfunction in DS is not surprising given that the DS thymus is small with an abnormal structure, even in the neonate, and shows a decreased proportion of phenotypically mature thymocytes [41–43]. T cell receptor (TCR) excision circle counts suggest that proportions of recent thymic emigrants in peripheral blood are decreased [44, 45]. Absolute numbers of T lymphocytes are also decreased in the DS neonate as well as the proliferative response to phytohemagglutinin, implying a deficient reaction to antigenic stimulation, but counts gradually approach those of normal children over time [46, 47]. Humoral immune-deficiencies have also been described in DS. In most of these reports, the number of B lymphocytes was found to be decreased and their ability to produce antibodies was diminished [46]. In addition, levels of CD4+ (T helper/inducer) were reduced and levels of CD8+ (T suppressor/cytotoxic) lymphocytes increased as compared with healthy subjects [46, 48, 49, 50]. As well as deficiencies in the adaptive immune response, the innate immune response is also altered in DS. In particular, it has been reported that natural killer (NK) cell frequency is altered and several studies have described a significant increase of cells possessing the low NK activity phenotype in DS, with an associated decrease of cells with the intermediate and high NK activity phenotype [51, 52].

Recent in-depth characterisations by Espinosa and colleagues suggest a state of chronic inflammation in DS, with consistent interferon overexpression and hyperactivation [53–55]. Proteomic analysis of DS plasma detected 299 proteins differentially regulated compared with controls with increased levels of proinflammatory cytokines such as IL-6, VEGF-A, MCP-1, IL-22 and TNF-α and downregulation of proteins involved in immune control [53•]. Interestingly, functional analysis of CD8+ and CD4+ T cells showed those with DS were less sensitive to T-reg suppression [54••]. Large-scale mass cytometry analyses did not link the myeloid compartment shift towards inflammation and over-activation of NK and cytotoxic T cells to existing comorbidities suggesting a high basal inflammatory environment in DS [55••]. Reduced sensitivity to regulation and an inflammatory state without a clinical diagnosis of autoimmunity suggests compromised central tolerance in DS. Interestingly, a type 1 interferon transcriptional signature has been shown to precede autoimmunity in children genetically at risk of type 1 diabetes [56]. The role of interferons in T1D has been well-reviewed by Lombardi et al. [57]. Taken together, observations of heightened inflammation in individuals with DS and those genetically at risk of type 1 diabetes may indicate a common mechanism underlying autoimmunity in DS.

### Environmental Factors in Down Syndrome

Children with Down Syndrome have a lower gestational age and a lower birth weight after 38 weeks compared to the general population [58]. Early birth has also been associated with T1D. Of 3,834,405 UK children under the age of 12 years, 2969 had a subsequent hospital diagnosis of type 1 diabetes in childhood. Children born preterm (<37 weeks) or early term (37-38 weeks) had a significantly higher incidence of subsequent type 1 diabetes than full term children (39-40 weeks) (HR 1.19 [95% CI 1.03, 1.38] and 1.27 [95% CI 1.16, 1.39], respectively). However low birthweight was not associated with future T1D, although higher birthweight was. This might suggest that factors associated with early birth but not birthweight may be important in DSD [59]. In studies of children genetically at risk for developing T1D, early life infections were associated with initiation of autoimmunty [60, 61]. Gastrointestinal infection and islet autoimmunty risk were dependent on feeding practices and weaning [61]. A large Norwegian general population study found that early life hopsitalisation with gastrointestinal infection increased risk of T1D but not infections not requiring hospitalisation or antibiotic use, [62]. In DS, children are more likely to be hosptialised due to infection and pneumonia is one of the major risk factors for motality [63, 64].

The development of the gut microbiome is modulated through early life feeding practices with the strongest differences seen in those who are breast-fed [65]. In children with early-onset T1D the gut micobiome has reduced diversity with an altered taxonomic profile [66]. Gut dysbiosis and immune barrier modulation with increased permeability was seen in a cohort of children with multiple islet autoantibodies [67]. The microbiome in DS has only been investigated in adulthood and was comparable to controls [68]. An important question remains on how differences in early life alter the gut microbiome in DS and how this, along with an immune system primed for inflammation could accelerate T1D.

### Genetic Strategies

**Fig. 1:**
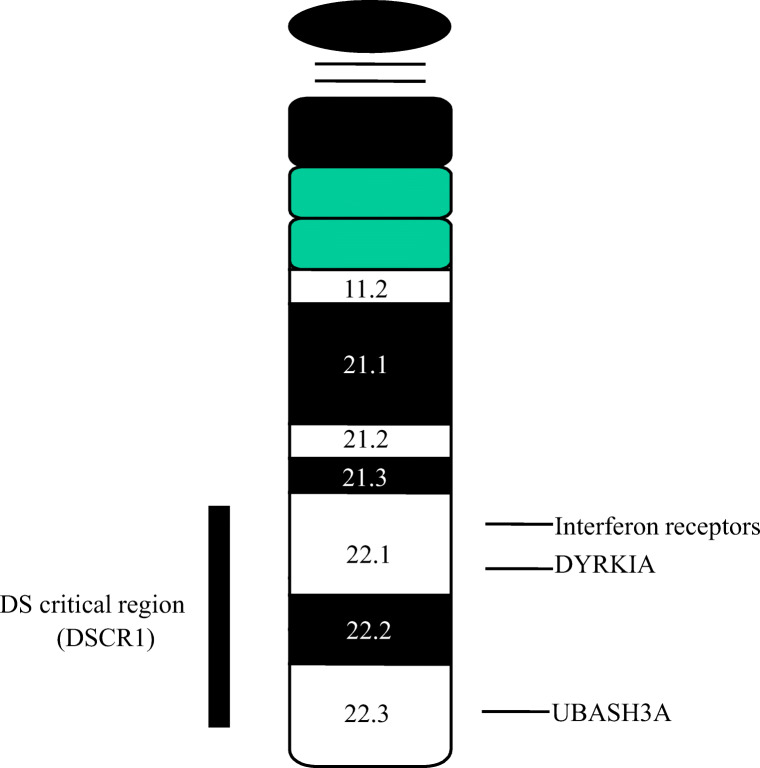
Chromosome 21. The Down’s syndrome critical region (DSCR) and genes that may be linked to the increased risk of autoimmunity in DS are indicated

Ninety-five percent of Down syndrome cases result from maternal non-disjunction with the remaining 5% caused by other chromosomal anomalies, particularly translocations. A region described as the Down syndrome critical region (DSCR) has been identified as key to the phenotypes associated with DS (Fig. 1). The study of individuals with partial trisomy 21 and other chromosome 21 rearrangements associated with clinical features of DS could identify genomic regions associated with specific phenotypes. The method of choice for high-resolution mapping to identify genomic rearrangements resulting in different copy numbers is array comparative genome hybridisation (aCGH). A study of 30 cases with different DS-related chromosome 21 rearrangements analysed by aCGH to refine genotype-phenotype mapping showed susceptibility regions for 25 clinical DS phenotypes (not including autoimmunity) [69].

Overexpression of candidate genes on chromosome 21 represents a plausible hypothesis for DS-associated phenotypes, but studies using gene expression arrays indicate that there is not an overall 50% increase in expression of chromosome 21 genes but rather that overexpression in different organs determines disease associations [70]. In the first extensive study of its kind [71], gene expression variation was analysed in 14 lymphoblastoid and 17 fibroblast cell lines from individuals with DS and an equal number of controls; 100 and 106 chromosome 21 genes, and 23 and 26 non-chromosome 21 genes respectively, were examined. Only 39% and 62% of chromosome 21 genes in lymphoblastoid and fibroblast cells, respectively, showed a significant difference between DS and normal samples. This emphasises the tissue-specific effects of gene dosage imbalance and indicates that each DS phenotype is likely to have a distinct mRNA expression “signature”.

In our earlier studies of Down syndrome and diabetes, data on clinical diagnosis of other autoimmune diseases were available on 92 subjects. Of these, 68 (74%) had coexisting thyroid disease and 11 (14%) had coexisting celiac disease. Seven of 92 (8%) had coexisting diagnoses of diabetes and thyroid and celiac disease. The frequency of HLA class II DRB1*03 was significantly increased in this group compared to 270 sex and age-matched children with type 1 diabetes alone (*p* = 0.002). One of the common pathways of autoimmunity in DS, therefore, appears to be mediated through HLA DR3 as has been shown for multiple autoimmunity in type 1 diabetes [72•]. A second is likely to be the Ubiquitin-associated and SH3 domain-containing A (*UBASH3A*). GWAS of 2496 multiplex T1D families from the Type 1 Genetics Consortium identified a chromosome 21 type 1 diabetes-associated locus 21q22.3 associated with T1D [73] which has been replicated [74]. This locus was also independently associated with susceptibility to coeliac disease (*P* = 0.009) [75]. The only gene in the corresponding region of linkage disequilibrium is *UBASH3A*, alternatively known as suppressor of T cell signalling 2 (STS-2), which comprises 15 exons, spans 40 kb and is expressed in spleen, bone marrow and peripheral blood lymphocytes including B cells and T cells [76]. To ensure that T cells are not inappropriately activated, signalling pathways downstream of the TCR are subject to multiple levels of positive and negative regulation. *UBASH3A* (STS-2) negatively regulates TCR signalling. Peripheral T lymphocyte activation in response to TCR/CD3 stimulation is known to be reduced in type 1 diabetic patients. *UBASH3A* is, therefore, a good candidate to contribute to the increased frequency of autoimmune disease including autoimmune diabetes in DS. Concannon and colleagues recently reported that *UBASH3A* attenuates NF-kB signal transduction upon TCR stimulation showing that *UBASH3A* is potentially causal in T1D [77•].

It has long been hypothesized that 3 copies of the chromosome 21 gene product *AIRE* that regulates ectopic expression of tissue-specific antigens in thymic medullary epithelial cells, crucial for thymic T cell selection, may underlie the increased frequency of autoimmunity in DS children, but this is counterintuitive. Rare mutations resulting in reduced function of Aire cause aggressive autoimmunity [78]. Analysis of protein and gene expression in surgically removed thymi from 14 DS patients compared with 42 age-matched controls has thrown some light on this conundrum. Results showed reduced expression of Aire in DS thymi [79], and this has been confirmed by other studies [78, 80].

As discussed earlier, type 1 interferon responses may pre-set the immune system towards autoimmunity in individuals with DS. Intriguingly, therefore, 4 of 6 interferon receptor subunits are encoded on chromosome 21 [81]. Although not identified as common variants associated with T1D in GWAS studies, they may be over-expressed in individuals with DS helping to create the underlying autoimmune environment. Further studies of these genes in DS and autoimmunity are warranted.

A gene on chromosome 21q22.13, within the DSCR, is dual-specificity tyrosine phosphorylation kinase 1a (*DYRK1A*) which catalyses its autophosphorylation on serine/threonine and tyrosine residues.

*DYRK1A* appears to play a key role in the signalling pathway that regulates cell proliferation including in brain development. While most research on this enzyme has focused on its potential role in cognitive impairment in DS, it has recently become clear that this kinase reciprocally regulates the differentiation of proinflammatory Th17 cells and regulatory T cells [82••], a crucial immunological balance in T1D. Chemical inhibition of *DYRK1A* with harmine enhanced differentiation of regulatory T cells (Tregs) from murine CD4+ naive T cells. Newly generated Tregs were able to suppress the proliferation of CD4+ effector cells stimulated with anti CD3/CD28. DYRK1A links mechanisms underlying cognitive and autoimmune impairments in DS.

## Conclusions

Many questions remain regarding the increased frequency of autoimmunity in DS. There is growing evidence for interferon hyperactivity which may mean that the immune balance is weighted towards autoimmunity even in young children with DS. It remains unclear why some develop autoimmunity and others do not. We have therefore initiated the Feeding and Autoimmunity in Down Syndrome Evaluation study (FADES) [83], a longitudinal birth cohort of children with DS, which will be key to determining early life impacts on risk of progression to autoimmunity in DS.

## Abbreviations

aCGH: Array comparative genome hybridisation
DS: Down syndrome
DSD: Down syndrome and diabetes
DSCR: Down syndrome critical region
GADA: Autoantibodies to glutamic acid decarboxylase
GWAS: Genome-wide association studies
HLA: Human leukocyte antigen
IAA: Insulin autoantibodies
IA-2A: Autoantibodies to IA-2
NK: Natural killer
STS-3: Suppressor of T cell signalling 2
T1D: Type 1 diabetes
TCR: T-cell receptor
UBASH3A: Ubiquitin-associated and SH3 domain-containing A
ZnT8A: Autoantibodies to zinc transporter 8
